# The Effect of Gabapentin on Acute Postoperative Pain in Patients Undergoing Total Knee Arthroplasty: A Meta-Analysis

**DOI:** 10.1097/MD.0000000000003673

**Published:** 2016-05-20

**Authors:** Lifeng Zhai, Zhoufeng Song, Kang Liu

**Affiliations:** From the Department of Orthopaedics (LZ), Tongde Hospital of Zhejiang Province; Department of Orthopaedics (ZS), Zhejiang Provincial Hospital of Traditional Chinese Medicine; and Department of Orthopaedics (KL), Second Hospital Affiliated to Zhejiang University of Chinese Medicine, Hangzhou, Zhejiang, China.

## Abstract

The purpose of this systematic review and meta-analysis of randomized controlled trials (RCTs) and non-RCTs was to evaluate the efficacy and safety of gabapentin versus placebo for pain control after total knee arthroplasty (TKA).

In December 2015, a systematic computer-based search was conducted in the Medline, Embase, PubMed, Cochrane Controlled Trials Register (CENTRAL), Web of Science, Google, and Chinese Wanfang databases. This systematic review and meta-analysis were performed according to the preferred reporting items for systematic reviews and meta-analyses (PRISMA) statement criteria. The primary endpoint was the visual analogue scale (VAS) score after TKA with rest or mobilization at 24 and 48 hours, representing the efficacy of pain control after TKA. Cumulative morphine consumption via patient controlled anesthesia (PCA) was also assessed to determine the morphine-spare effect. Complications such as dizziness, pruritus, vomiting, nausea, and sedation were also compiled to assess the safety of gabapentin. Stata 12.0 software was used for the meta-analysis. After testing for publication bias and heterogeneity across studies, the data were aggregated for random-effects modeling whenever necessary.

Six studies involving 769 patients met the inclusion criteria. Our meta-analysis revealed that gabapentin resulted in superior pain relief compared to the control group in terms of VAS score with rest at 24 hours (mean difference [MD] = −3.47; 95% confidence interval [CI] −6.16 to −0.77; *P* = 0.012) and at 48 hours postoperatively (MD = −2.25; 95% CI −4.21 to −0.30; *P* = 0.024). There was no statistically significant difference between the groups with respect to the VAS score at 24 hours postoperatively (MD = 1.05; 95% CI −3.31 to 5.42; *P* = 0.636) or at 48 hours (MD = 1.71; 95% CI −0.74 to 4.15; *P* = 0.171). These results indicated that the perioperative administration of gabapentin decreases the cumulative morphine consumption via PCA at 24 hours (MD = −8.28; 95% CI −12.57 to −3.99; *P* = 0.000) and 48 hours (MD = −4.50; 95% CI −10.98 to −3.61; *P* = 0.221). Furthermore, gabapentin decreased the rate of postoperative dizziness (relative risk [RR], 0.68; 95% CI 0.47–0.99, *P* = 0.044) and the occurrence of pruritus (RR, 0.50; 95% CI 0.37–0.67, *P* = 0.000).

Based on the current meta-analysis, gabapentin exerts an analgesic and opioid-sparing effect in acute postoperative pain management without increasing the rate of dizziness and pruritus.

## INTRODUCTION

Total knee arthroplasty (TKA) is one of the most common surgeries performed on patients with osteoarthritis or rheumatic arthritis of the knee. However, TKA is associated with moderate to severe pain after the operation.^1^ Resuming ambulation as soon as possible after the operation can decrease the occurrence of deep venous thrombosis and the economic cost of recovery.^2^ However, the pain following TKA is especially intense during mobilization, thus, peripheral nerve block, liposomal bupivacaine, and oral morphine have been used to reduce the severity pain after TKA. Pang et al^3^ reported that the pain occurring after TKA is more painful than that of any other orthopedic surgery, including total hip arthroplasty. The mechanism of postoperative pain involves the sensitization of peripheral nociceptive nerve terminals and central neurons.^4^ Recently, the sensitization of central neurons have been demonstrated to be more important than peripheral nerve sensitization.

Contemporary postoperative pain management is aimed at enhancing pain relief and decreasing opioid consumption by combining analgesic drugs and techniques to reduce opioid-related complications. A variety of modalities have been applied to reduce postoperative pain after TKA, including intravenous patient-controlled epidural analgesia with opioids, local infiltration analgesia with levobupivacaine, ketorolac and adrenaline, and gabapentin.^5,6^ The use of opioids is limited by adverse effects such as nausea, vomiting, and pruritus.^7^ Woolf and Chong^8^ have proposed that antihyperalgesic drugs such as gabapentin and pregabalin added to the multiple anesthesia can be used to reduce severe pain and morphine consumption via patient-controlled anesthesia (PCA) after TKA. Recently, many studies have compared gabapentin with a placebo to manage pain after TKA. However, the results of these studies are contradictory. There is no consensus regarding the efficacy of gabapentin for managing TKA pain. We therefore searched electronic databases and conducted a systematic review and meta-analysis to identify the clinical outcome and safety of gabapentin in reducing pain after TKA.

## MATERIALS AND METHODS

This review is registered in Protocol registration: PROSPERO 2015: CRD42015032298.

### Search Strategy

The following electronic databases were searched for relevant academic clinical trials comparing perioperative gabapentin to a placebo for the management of pain after TKA from inception to December, 2015: Medline, Embase, PubMed, Cochrane Controlled Trials Register (CENTRAL), Web of Science, Chinese Wanfang, and Google. The key words and medical subject heading terms included the following: gabapentin, pain control, total knee arthroplasty, total knee replacement, TKA, and TKR. These key words and the corresponding medical subject heading terms were combined with the Boolean operators AND and OR. Furthermore, the reference lists of the identified literature were reviewed to identify any initially omitted studies, and no restriction was made on the language of the publication. Two reviewers (LZ and ZS) independently searched the databases and filtered the relevant literature. Conflicts were resolved by the 3rd reviewer (KL). The full articles were screened to determine whether the articles fit the inclusion and exclusion criteria. As this is a meta-analysis, no ethics committee or institutional review board approval was required.

### Inclusion Criteria and Study Selection

The inclusion criteria were as follows: randomized controlled trials (RCTs) and non-RCTs; patients who underwent a primary TKA; interventions, including gabapentin with a control (placebo or nothing); and reported outcomes, including postoperative visual analogue scale (VAS) pain with rest or mobilization at 24 and 48 hours and active knee flexion at days 2 and 3, as well as the incidence of pruritus, vomiting, dizziness, sedation and nausea, and cumulative morphine consumption via PCA at 24 and 48 hours. The article needed to include at least one of the outcomes mentioned above. We excluded studies of cadavers or artificial models. We also excluded non-RCTs, letters, comments, editorials, practice guidelines, and other studies with insufficient data.

### Data Abstraction and Quality Assessment

Duplicates were excluded using Endnote software, and 2 reviewers independently screened the titles and abstracts of the searched literature. Most of the articles were excluded based on the topic of the article provided in the title or abstract, and disagreements about whether or not an article should be included were resolved by discussion or by a senior reviewer. Postoperative pain intensity was measured on a 100-point VAS. The 10-point VAS score was converted to a 100-point VAS score. Data in other forms (i.e., median, interquartile range, and mean ± 95% confidence interval [CI]) were converted to mean ± SD according to the Cochrane Handbook.^9^ If the data were not reported numerically, we extracted them using the “GetData Graph Digitizer” software from the published figures.

The following data were extracted and recorded in a spreadsheet: the author's name, demographic data about the number of patients in the gabapentin and control groups, the number of male patients in each group, the dose and time to administration of gabapentin, and the anesthesia method; intraoperative and postoperative analgesia; and the VAS score with rest or mobilization at 24 and 48 hours, the rates of pruritus, vomiting, dizziness, sedation and nausea, and the cumulative morphine consumption via PCA at 24 and 48 hours. Two reviewers independently scanned the quality of the eligible studies. Discrepancies were resolved by consensus after discussion, and a 3rd reviewer participated in the debate to determine the final outcome if necessary. The risk of bias for each RCT was evaluated using the Cochrane Collaboration's Risk of Bias Tool.

### Statistical Analysis

Continuous outcomes such as the VAS score with rest or mobilization at 24 and 48 hours, the morphine cumulative consumption via PCA at 24 and 48 hours, and active knee flexion were expressed as the mean difference (MD) with the respective 95% CIs. Discontinuous outcomes (i.e., the rate of pruritus, vomiting, dizziness, sedation, and nausea) were expressed as the relative risk (RR) with 95% CIs. Statistical significance was set at *P* < 0.05 to summarize the findings across trials. RevMan 5.30 software (The Cochrane Collaboration, Oxford, United Kingdom) was used for the meta-analysis. Different gabapentin doses and dosing times in a single study were handled as subgroups within the study. Statistical heterogeneity was tested using the Chi-squared test and *I*^2^ statistic. A Chi-squared test scoring *I*^2^ > 50% was considered suggestive of statistical heterogeneity. When there was no statistical evidence of heterogeneity, a fixed effects model was adopted; otherwise, a random effects model was chosen.

## RESULTS

### Search Results

**FIGURE 1:**
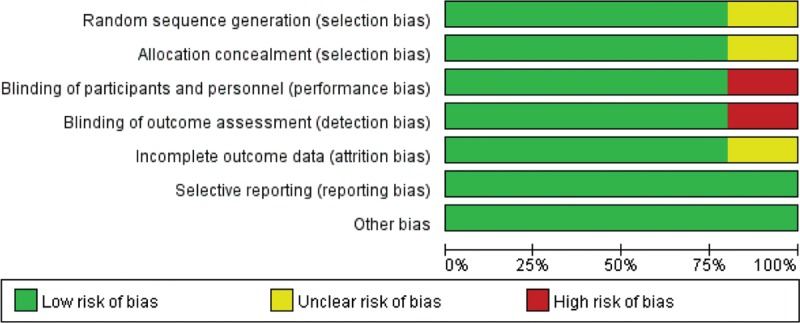
The summary of bias of the included studies.

**FIGURE 2:**
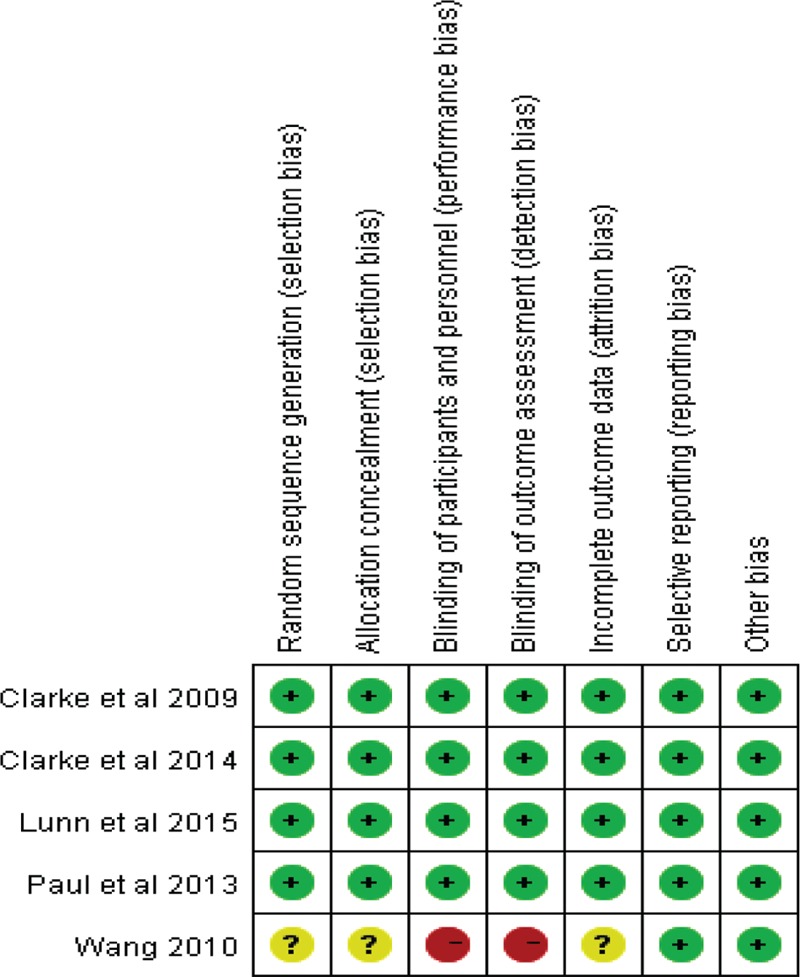
The bias of each included studies.

In the initial search, we identified 312 potentially relevant studies, of which 30 duplicates were removed by Endnote Software. According to the inclusion criteria, 276 studies were excluded after reading the titles and abstracts. Finally, we included 6 clinical trials with 769 patients in the meta-analysis.^10–15^ In the included studies, 2 articles were produced by the same team; however, after carefully reading the literature, we determined that the methods and the patients were different. Therefore, both studies were included in our analysis. Among the included studies, 5 articles were RCTs and 1 study was a non-RCT. The characteristics of the included studies are shown in Table 1, and the dose and time to administration of gabapentin are shown in Table 2. In the included studies, a total of 769 TKAs were performed, and the number of studies using gabapentin analgesia and a control group were 451 and 318, respectively. Two articles were published in 2009;^12,13^ 1 was published in 2010,^10^ and the others were published in 2010.^11,14–16^ The participants in the 5 studies were mostly elderly, but one study included young patients. The age of the patients ranged from 36 to 70 years old. There were 263 male patients and 298 female patients. The plasma drug concentration peaked at 2∼3 h after oral the gabapentin and elimination half-life for normal patients is 4.8–8.4 h. The dose of gabapentin ranged from 400 mg/kg to 600 mg/kg preoperatively and from 200 mg/kg to 400 mg/kg postoperatively. The intraoperative analgesia consisted of local infiltration, general analgesia and spinal analgesia. The postoperative analgesia included acetaminophen, celecoxib, PCA, non-steroidal anti-inflammatory drugs, and morphine or celecoxib; details can be seen in Table 2. All five RCTs introduced randomization; only one trial did not imply random sequence generation. One study did not introduce concealment. The risk of bias can be seen in Figures 1 and 2.

**TABLE 1:**
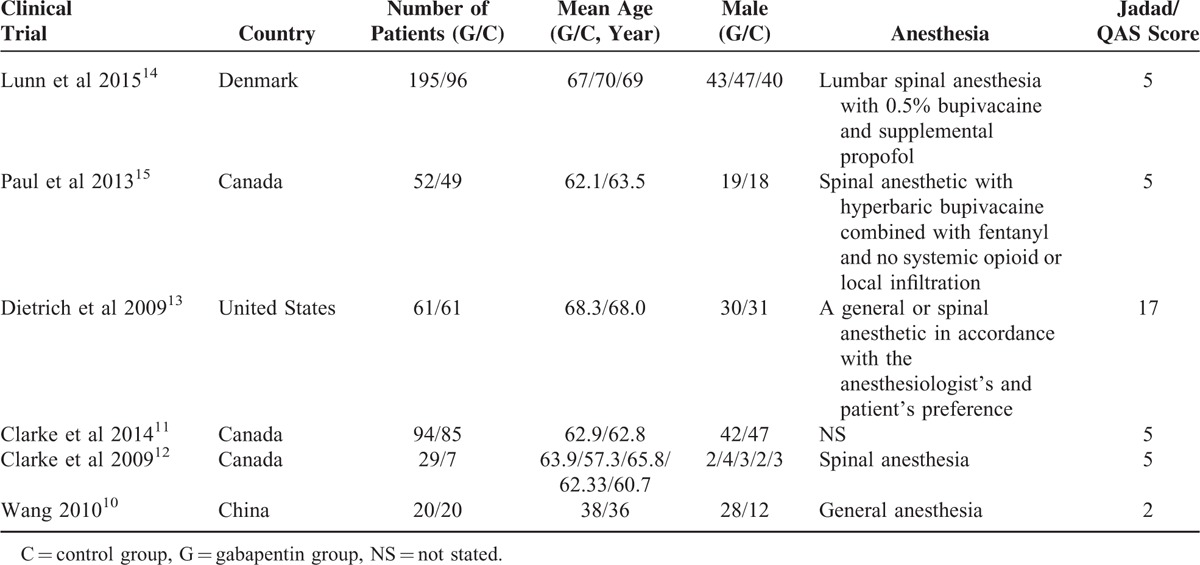
The General Characteristic of the Included Studies

**TABLE 2:**
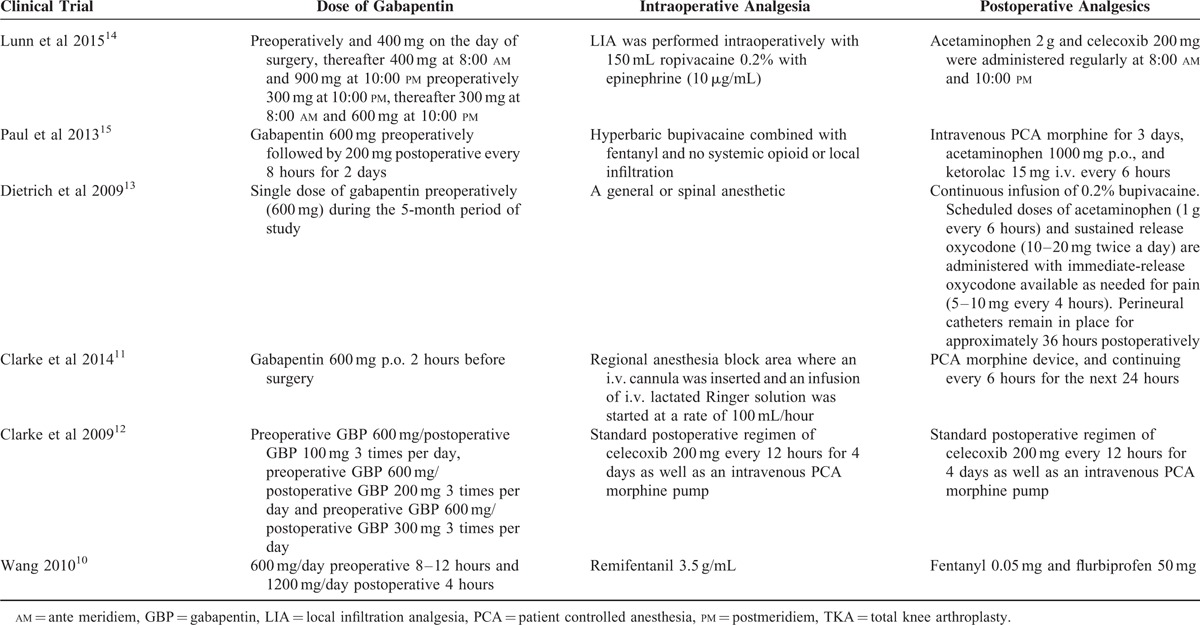
The Methods That Management the Pain During TKA and Postoperative

### Results of the Meta-Analysis

#### VAS Score With Rest

**FIGURE 3:**
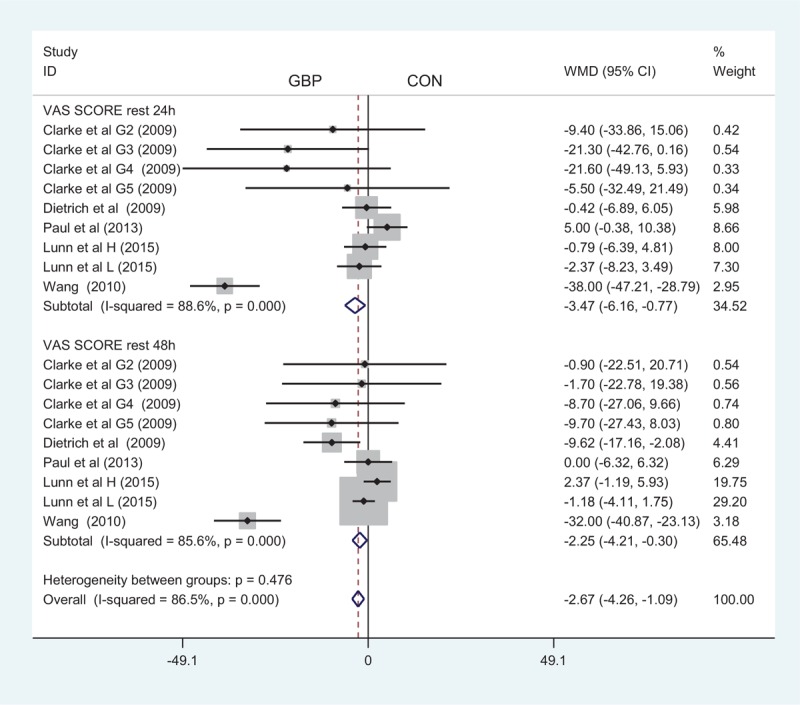
The forest plot of visual analogue scale (VAS) score with rest at 24 and 48 hours.

Only 9 studies with 717 patients provided a VAS score at 24 hours after surgery with rest. Among these studies, 1 administered gabapentin at different doses versus a placebo, so a total of 9 clinical studies were included. Our meta-analysis revealed that gabapentin produced a better outcome compared to the control group with rest at 24 hours in terms of VAS score (MD = −3.47; 95% CI −6.16 to −0.77; *P* = 0.012, Figure 3).

A total of 9 component studies (717 patients) provided VAS scores at 48 hours postoperatively. There was a statistically significant difference between the groups with respect to the VAS score at 48 hours postoperatively (MD = −2.25; 95% CI −4.21 to −0.30; *P* = 0.024, Figure 3).

**FIGURE 4:**
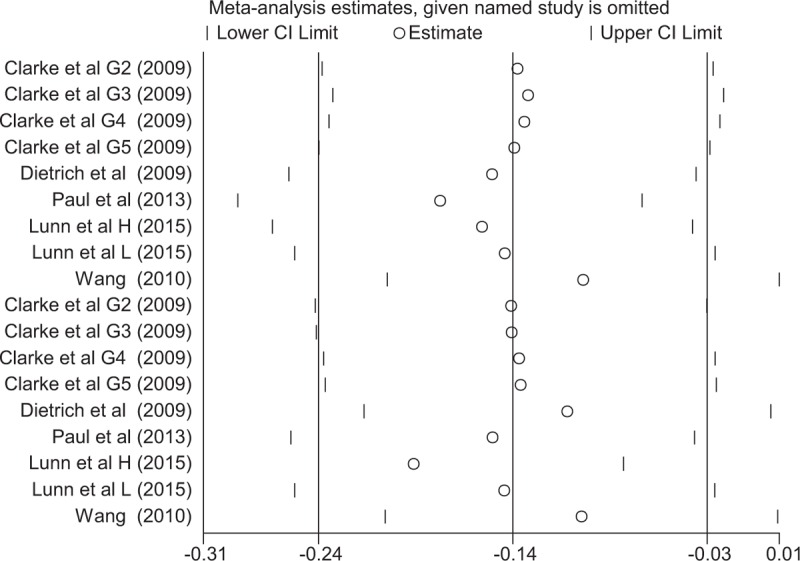
The sensitivity analysis of the visual analogue scale (VAS) score at 24 and 48 hours with rest.

To determine the source of heterogeneity and to enhance the credibility of our results, a sensitivity analysis was conducted. Based on the result of a sensitivity analysis, Wang et al^10^ showed a remarkable influence on heterogeneity (Figure 4). Wang's study^10^ included 40 patients and did not manage pain postoperatively, which may account for the difference from the other studies.

### VAS Score With Mobilization

**FIGURE 5:**
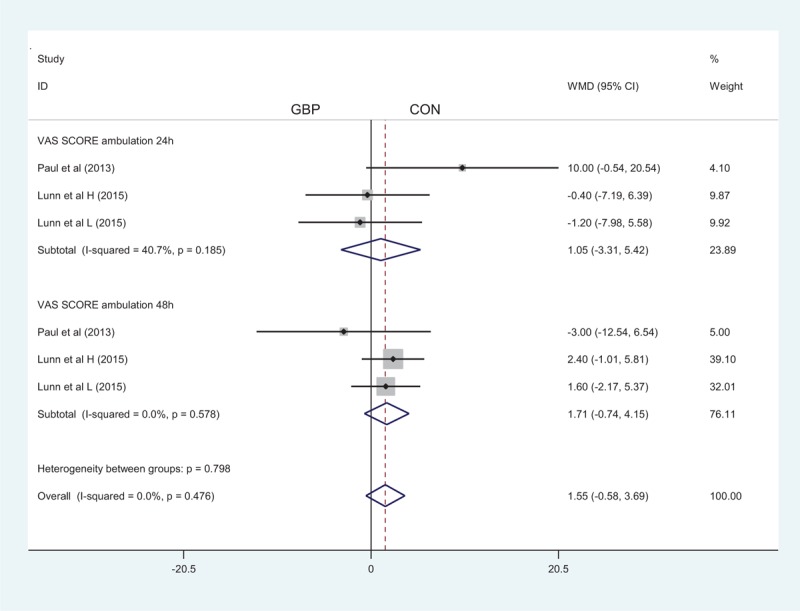
The forest plot of visual analogue scale (VAS) score with ambulation at 24 and 48 hours.

A total of 3 component studies (498 patients) provided VAS scores at 24 hours with mobilization postoperatively. There was no statistically significant difference between the groups with respect to the VAS score at 24 hours postoperatively (MD = 1.05; 95% CI −3.31 to 5.42; *P* = 0.636, Figure 5). Only 3 studies with 498 TKAs reported the VAS score at 48 hours postoperatively; our meta-analysis found no significant difference between the 2 groups (MD = 1.71; 95% CI −0.74 to 4.15; *P* = 0.171, Figure 5).

### Cumulative Morphine Consumption via PCA ay 24 and 48 Hours

**FIGURE 6:**
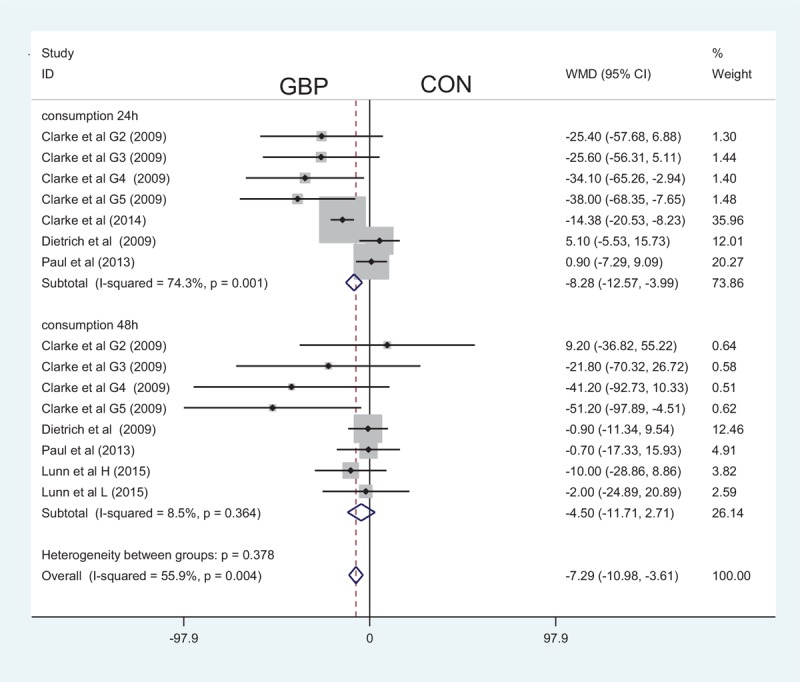
The forest plot about the effect of gabapentin on the cumulative morphine consumption at 24 and 48 hours.

A total of 7 studies and 8 articles addressed the cumulative morphine consumption via PCA at 24 and 48 hours, respectively, between the gabapentin and the control groups. The results indicated that perioperative gabapentin can decrease the cumulative morphine consumption via PCA at 24 hours (MD = −8.28; 95% CI −12.57 to −3.99; *P* = 0.000, Figure 6) and 48 hours (MD = −4.50; 95% CI −10.98 to −3.61; *P* = 0.221, Figure 6). However, there was statistically significant heterogeneity between the studies, and sensitivity analysis and publication bias measures were taken to diminish these effects.

### Complications

**FIGURE 7:**
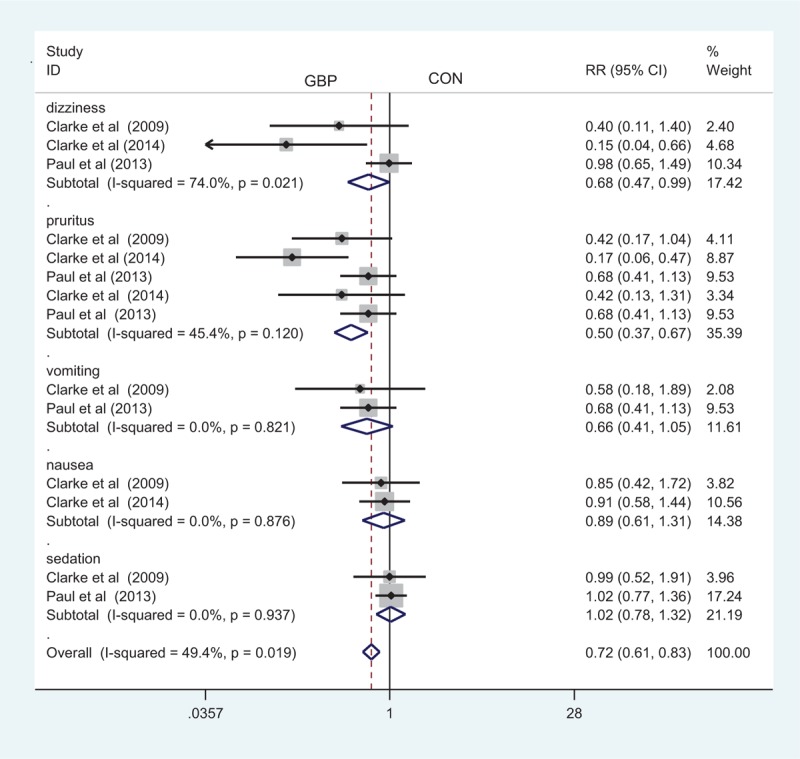
The forest plot about the occurrence of gabapentin on the dizziness, pruritus, vomiting, nausea, and sedation.

Three studies paid close attention to postoperative dizziness. Our meta-analysis identified a significant difference between the 2 methods in terms of postoperative dizziness (RR, 0.68; 95% CI 0.47–0.99, *P* = 0.044, Figure 7), with a high heterogeneity. Five studies investigated the occurrence of pruritus in both methods and found that the administration of gabapentin can decrease the occurrence of pruritus (RR, 0.50; 95% CI 0.37–0.67, *P* = 0.000, Figure 7). In addition to the above complications, there was no statistically significant difference between the rates of vomiting, nausea, and sedation (RR, 0.66; 95% CI 0.41–1.05, *P* = 0.081, RR, 0.89; 95% CI 0.61–1.31, *P* = 0.571, RR, 1.02; 95% CI 0.78–1.32, *P* = 0.898, Figure 7).

## DISCUSSION

To our knowledge, this is the 1st meta-analysis of RCTs and non-RCTs comparing the efficacy and safety of gabapentin with a placebo for the management of pain after TKA. The present meta-analysis was conducted on the basis of 5 randomized studies and 1 non-RCT that found better pain control with rest at 24 and 48 hours postoperatively with gabapentin administration compared to controls. There was no significant difference between the 2 groups with mobilization at 24 or 48 hours. In addition, perioperative gabapentin administration can decrease the occurrence of dizziness and pruritus. It has been established that pain control with mobilization results in greater benefits than rest because mobilization can decrease the occurrence of deep venous thrombosis. Two articles were published in 2009 and the others were all published from 2010. Four included studies were of high quality; only 1 study had a low score of Jadad. The other non-RCT was also a high-quality article; it was a matched pair study, and the general character between the 2 groups exhibited no significant difference. All of the included studies presented comparable baseline data and provided an intention to treat analysis. Only 1 study included young patients to perform TKA.^10^ And the total number of patients needed to treat is 769, the number of the gabapentin group and control group is 451 and 318, respectively.

The pain after TKA is typically severe, making good pain management especially important for early mobilization to decrease the length of the hospital stay.^17–19^ Multimodal analgesic pathway has been recommended to decrease severe pain after TKA for 25 years, and the objective of multimodal postoperative analgesia is to decrease pain intensity and therefore reduce consumption and opioid-related complications. Gabapentin was first introduced as an antiepileptic drug in 1993 and has since been used to treat painful neuropathies. The effect of gabapentin on pain relief differs between surgical patients; Dahl et al^20^ reported that there was no significant reduction in opioid consumption during the first 24 hours in 6 of 7 studies. Wiffen et al^21^ reported that there is no role for gabapentin in the management of chronic and acute pain.

The effect of adjunct gabapentin on multimodal postoperative analgesia is controversial. The results of our meta-analysis showed that gabapentin resulted in improved pain relief compared to the placebo in combination with rest at 24 and 48 hours postoperatively; however, there was no significant difference in pain relief accompanied by mobilization at 24 and 48 hours. The main mechanism of gabapentin action is achieved in combination with the 21 subunits of presynaptic voltage-gated calcium channels. The expression of these channels is upregulated upon nerve injury. Furthermore, gabapentin can decrease the hyperexcitability of secondary nociceptive neurons in the dorsal horn. As for the lack of efficacy at 24 and 48 hours when accompanied by mobilization, the number of patients involved in the comparison of VAS score with mobilization was limited – only 3 studies reported this outcome. This is in contrast to a study by Dirks et al,^22^ who compared the effect of a single dose of gabapentin with a placebo on reducing postoperative pain after mastectomy. This study found that gabapentin can decrease pain with mobilization. Hwang et al^23^ conducted a meta-analysis to compare the effect of gabapentin in the management of pain relief after tonsillectomy and found that gabapentin provided pain relief without side effects.

As for cumulative morphine consumption via PCA, gabapentin decreased the cumulative morphine consumption at 24 and 48 hours; however, there was no statistically significant difference between the 2 groups at 48 hours. This result contradicts that of Tiippana 2007 meta-analysis;^24^ in the latter study, all postoperative pain was managed with gabapentin, suggesting that gabapentin markedly reduces opioid consumption and enhances pain relief. Doleman et al^25^ conducted a systematic review and meta-regression analysis to evaluate the use of prophylactic gabapentin for the management of postoperative pain and found that gabapentin can decrease the mean morphine consumption by approximately 8.44 g. Paul et al^26^ conducted an RCT to compare the effect of gabapentin on reducing morphine consumption at 72 hours; the result of this study indicated that gabapentin exhibited no clinically important reduction in postoperative morphine consumption at 72 hours.

The occurrences of pruritus and dizziness were significantly lower in the gabapentin group, and the difference was statistically significant. This can be explained by the lower morphine consumption of patients receiving gabapentin and the associated decrease in the adverse effects of opioids. There were also lower rates of vomiting, nausea, and sedation; however, there was no significant difference between the 2 groups. In theory, gabapentin may result in more dizziness than a placebo, but the result of this meta-analysis indicated that the incidence of dizziness in the gabapentin group was lower than that in the placebo group. Ho et al^27^ conducted a meta-analysis to compare the effect of gabapentin on reducing postoperative pain and found that gabapentin increased the occurrence of dizziness. In addition, a higher incidence of dizziness was reported in a study by Lunn et al^14^ in gabapentin versus control groups; however, the incidence in each group was not reported separately, and the data did not agree with our analysis, thus affecting our conclusions.

There were several limitations in this meta-analysis: only 5 RCTs and 1 non-RCT were included, and the sample sizes in the 2 trials were small, which would affect the final results; the duration of follow-up in some studies was unclear, and long-term follow-up was needed for this analysis; the publication bias that existed in the meta-analysis also influenced the results; and the dose and time of gabapentin differed between studies, which will affect the precision of the result.

## CONCLUSION

In conclusion, although the number of studies and samples in each paper is limited, this is the 1st meta-analysis that compares the use of gabapentin with a placebo for the management of pain after TKA. Based on our meta-analysis, gabapentin has an analgesic- and opioid-sparing effect in acute postoperative pain management without increasing the rate of dizziness and pruritus. As the sample size and number of included studies are limited, a multiple central randomized controlled trial is still needed to identify the effect and the optimal dose of gabapentin in for reducing pain after TKA.
